# Cross-species testing and utility of microsatellite loci in *Indirana* frogs

**DOI:** 10.1186/1756-0500-5-389

**Published:** 2012-07-29

**Authors:** Abhilash Nair, Sujith V Gopalan, Sanil George, K Santhosh Kumar, Juha Merilä

**Affiliations:** 1 Ecological Genetics Research Unit, Department of Biosciences, University of Helsinki, PO Box 65, Helsinki, FI-00014, Finland; 2 Chemical Biology, Rajiv Gandhi Centre for Biotechnology, PO Thycaud, Poojappura, Thiruvananthapuram, Kerala, 695 014, India

**Keywords:** Amphibia, Microsatellite, *Indirana*, Biodiversity hotspot, Ranixalidae, Western Ghats

## Abstract

**Background:**

Microsatellite loci are widely used in population and conservation genetic studies of amphibians, but the availability of such markers for tropical and subtropical taxa is currently very limited. In order to develop resources for conservation genetic studies in the genus *Indirana*, we tested amplification success and polymorphism in 62 previously developed microsatellite loci, in eight *Indirana* species - including new candidate species. Developing genomic resources for this amphibian taxon is particularly important as it is endemic to the Western Ghats biodiversity hotspot, and harbours several endangered species.

**Findings:**

The cross-species amplification success rate varied from 11.3 % to 29.0 % depending on the species, with 29 - 80 % of the amplifying loci being polymorphic. A strong negative correlation between cross-species amplification success (and polymorphism) and genetic distance separating target from source species was observed.

**Conclusions:**

Our results provide additional genetic support for the existence of genetically divergent cryptic species within the genus *Indirana*. The tested markers should be useful for population and conservation genetic studies in this genus, and in particular, for species closely related to the source species, *I. beddomii*.

## Background

The fauna of the Indian Western Ghats biodiversity hotspot is well known for its diversity and high level of endemism [1]. In particular, there are many families and genera of amphibians that are unique to the region [2,3], with roughly 132 endemic species [4]. However, the amphibian diversity in this region remains inadequately characterized [5]. This is reflected by numerous taxonomic uncertainties and ambiguities [6], and in the fact that many species still await proper taxonomic description [7]. Frogs belonging to the endemic genus *Indirana* (*Ranixalidae*; [8]) are among the poorly studied amphibian genera from the Western Ghats [9]. At present, information regarding their interspecific and intraspecific differentiation and variability across the diverse and fragmented habitats of Western Ghats is very limited [10].

An earlier study based on the examination of interspecific mitochondrial and nuclear sequence variability revealed a great deal of cryptic diversity within the *Indirana* genus; morphologically similar species displayed a high degree (4.2–17.1 %) of genetic divergence [10]. In order to further explore this differentiation, we have recently developed 62 polymorphic microsatellite loci for one of the *Indirana* species (*I. beddomii*; [11]). Here we report the results of cross-species amplification tests for these microsatellite loci in eight other species (including candidate species) within the *Indirana* genus. Apart from providing information about the cross-species utility of these markers, we also investigated how the amplification success and levels of polymorphism relate to the evolutionary divergence between target and source species.

## Methods

The samples were collected during field surveys in the southern Western Ghats between 2008 and 2010, under licence from National Biodiversity Authority, India (licence #NBA/TECH Appl/9/85/34/08/08-09/682).

The 62 microsatellite markers to be tested were developed using samples of *I. beddomii* from southern Kerala (08°45’59”N, 77°06’34”E; [11]). However, specimens thought to be *I. beddomii* from sites in northern and central Kerala (Aralam 11°55’54”N, 75°50’09”E; and Periyar 09°29’27”N, 77°08’10”E) and Kudremukh in Karnataka (13°12’38”N, 75°11’19”E) have previously shown high genetic divergence (4.2-12.5 %) from *I. beddomii* frogs from southern Kerala, and from each other [10]. This high genetic divergence suggested that these were cryptic species albeit morphologically similar to each other and to *I. beddomii*. Therefore, *I*. *beddomii* as currently recognized appears not to be a single species, but a complex of at least four distinct species [10]. The same also seems to apply to another species, *I. diplosticta*[10]. We tested for cross-species microsatellite amplification in four putative species, along with three known species in the genus (*I. semipalmataI. diplostictaI. leptodactyla*), and in an additional unknown species (*Indirana* sp., Additional file 1). Each primer pair developed for *I*. *beddomii* (the “source” species) was tested against all of the “target” species and scored for amplification success and polymorphism. Cross-species amplification tests were performed using 5–10 individuals for each of the eight species/new candidate species within the genus *Indirana* (Additional file 1).

DNA was extracted from toe clips using a DNeasy Blood and Tissue kit (QIAGEN). Forward primers were labelled with fluorescent dyes FAM, HEX and TET (DNA Technology A/S), and a 5'-GTTT “tail” was added to every reverse primer to facilitate accurate genotyping [12]. PCR reactions were performed for each primer pair in a total reaction volume of 10μL consisting of 1× QIAGEN Multiplex PCR solution, 0.2-0.3 μM of primers, dH_2_O and 10–20 ng of template DNA. The following PCR cycling conditions were used for amplification: 95 °C for 15 min, followed by 30 cycles of 95 °C for 30 s, 55 °C for 1.5 min, 72 °C for 1 min, and then a final extension step at 60 °C for 10 min. The PCR products were diluted 1:100 and electrophoresed on a MegaBACE 1000 capillary sequencer with MegaBACE ET550-R size standard. Genotypes were scored using Fragment Profiler (ver. 1.2; GE Healthcare Life Sciences). The amplification was considered positive if one or two alleles were observed with little or no stutter in comparison to a positive control. Decreasing the PCR annealing temperature is known to increase amplification success and polymorphism detection in cross-species amplification tests [13]. Therefore, markers that did not amplify initially were re-tested for amplification at 52 °C. Annealing temperatures lower than 52 °C were not used, as they have not proved to be useful in identifying polymorphic loci in earlier cross-species tests [14].

The mitochondrial 16S ribosomal RNA gene sequences (466 bp) of *Indirana* species were retrieved from Genbank [Genbank: JQ596642-44, JQ596648-85]. We used 3–5 sequences per species to estimate the genetic distance between species (i.e. source-target species divergence) based on Kimura two parameter model [15] using MEGA5 [16]. All statistical tests were performed using the program SPSS 15 (SPSS Inc., Chicago IL, USA). The species identity of the individuals used in the study was ascertained using 16S sequence as barcodes, following methods described in Nair et al. [10].

## Results

**Figure 1:**
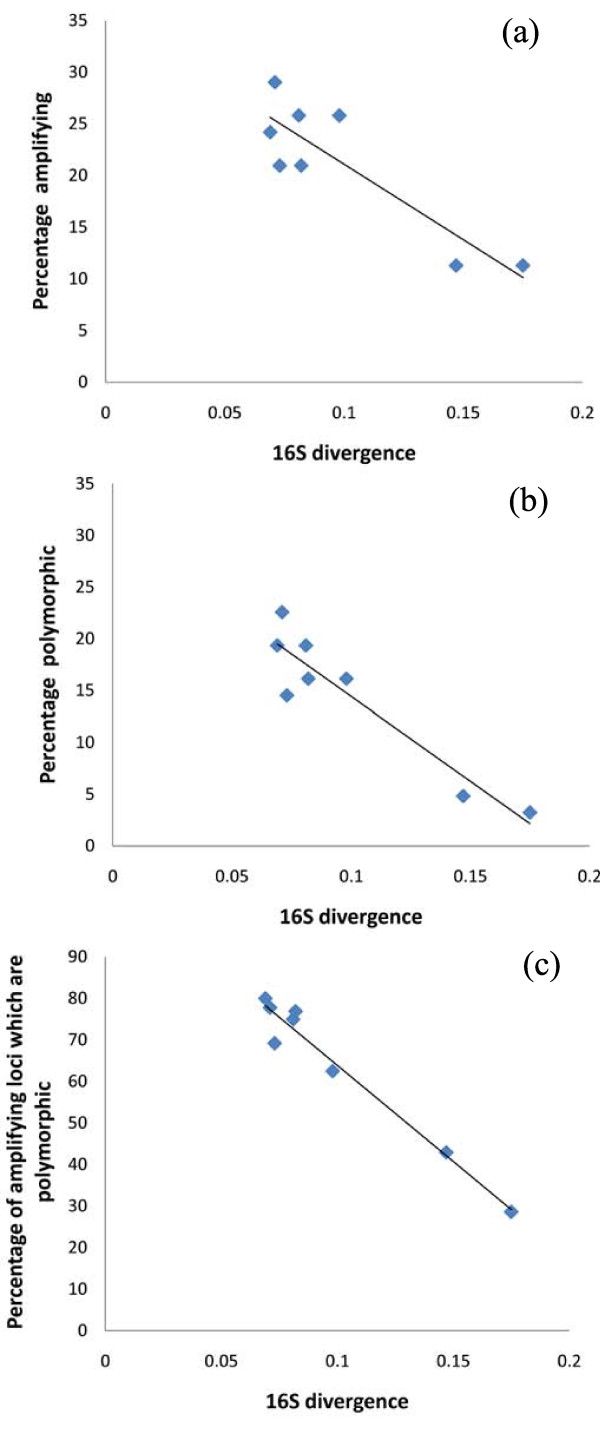
**Relationship between genetic divergence and cross species amplification success.** Relationship between (**a**) percentage of amplifying loci, (**b**) percentage of polymorphic loci and (**c**) percentage of amplifying loci which are polymorphic, and the mitochondrial 16S divergence from the source species.

The cross-species amplification tests resulted in successful amplification of 7–18 loci (11.3 – 29.0 %) depending on the species analysed, and 2–14 of these loci were polymorphic in the target species (3.2 - 22.6 %; Table 1). Two of the monomorphic loci in the source species *I*. *beddomii* (IND 11, IND 15) were polymorphic in at least one of the other species, whereas locus IND 24 was monomorphic in all the species (Additional file 1). The genetic divergence between source and target species in 16S sequence ranged from 6.9 to 17.5 %. The extent of the cross species amplification success (*r* = −0.87, *r*^*2*^ = 0.76, *P* < 0.01; Figure 1a), proportion of the polymorphic loci (*r* = −0.94, *r*^*2*^ = 0.88, *P* < 0.01; Figure 1b) and polymorphism among amplifying loci (*r* = −0.98, *r*^*2*^ = 0.96, *P* < 0.01, Figure 1c) were strongly negatively correlated with genetic divergence between the target and source species. Lowering the annealing temperature for those initially non-amplifying markers did not result in amplification of any additional loci.

**Table 1:** **Cross-species amplification success rate of the 62 microsatellite loci tested on*Indirana*species**

**Species**	**16S divergence**	**No. of individuals tested**	**No. of amplifying loci**	**% amplifying**	**No. of polymorphic loci**	**% polymorphic**	**% of amplifying loci which are polymorphic**
*I. semipalmata*	0.069	10	15	24.2	12	19.3	80.0
Indirana sp*	0.071	10	18	29.0	14	22.6	77.8
*I. beddomii* * (Aralam)	0.073	10	13	21.0	9	14.5	69.2
I. beddomii* (Kudremukh)	0.081	10	16	25.8	12	19.3	75.0
I. diplosticta* (Vellarimala)	0.082	10	13	21.0	10	16.1	76.9
*I. beddomii* * (Periyar)	0.098	10	16	25.8	10	16.1	62.5
*I. leptodactyla*	0.147	10	7	11.3	3	4.8	42.9
*I. diplosticta*	0.175	5	7	11.3	2	3.2	28.6

*new candidate species according to Nair et al. [10].

## Discussion

It is well known that the utility of the microsatellites developed for a particular species is a negative function of the genetic distance separating the target and source species [13,17,18], which has been shown in birds, cetaceans, frogs [13] and other amphibians (e.g. [19]). Our results from the *Indirana* genus conform to these general patterns, and the observed cross-species amplification success rate of 11.3-29.0 % (mean = 21.2 %) is comparable to the within-genus amplification success rate of approximately 21 % reported for Ranid frogs [20]. The fact that we found relatively low cross-species amplification success rates of 21.0 %, 25.8 %, and 25.8 % for the three cryptic species that were previously thought to be *I. beddomii*, supports the idea – based on analyses of multiple nuclear and mitochondrial genes [10] – that the Aralam, Kudremukh and Periyar populations of what was thought to be *I. beddomii* are indeed distinct species [10].

One of the main reasons for lowered cross-species amplification success is thought to be the lack of conservation of priming sites between highly divergent species [21]. Additionally, increased genome size (C-value) is also known to have a negative effect on cross-species amplification success [22,23]: a decrease in the ratio of target to non-target DNA causes a reduction in amplification efficiency [24,25]. Regardless of the proximate cause, the close correspondence between the proportion of amplifying loci and 16S divergence among the tested taxa (Figure 1a) – as well as the proportion of polymorphic loci and 16S divergence (Figure 1b & c) – suggests comparable rates of divergence of mitochondrial and nuclear genomes in these frogs. While these patterns are admittedly driven by low amplification success in the two highly divergent species, *I*. *diplosticta* and *I*. *leptodactyla* (14.7-17.5 %)*,* the data nonetheless suggests that the cross-species utility of these markers declines with increasing evolutionary distance among taxa. For example, only 2–3 out the 62 tested loci were found to be polymorphic in the two most divergent taxa, limiting the utility of these markers in studies of divergent *Indirana* species. Interestingly, one of the loci (IND107) was highly polymorphic in all taxa studied, with similar numbers of alleles observed both in the source and most divergent species (Additional file 1). This locus appears to be conserved in all species within *Indirana* and could be associated with some functionally important gene. Conservation of microsatellite loci residing within or close to functionally important genes has been reported from other highly divergent species (e.g. [26,27]).

Taken together, the results of this study show that the cross-species amplification success in *Indirana* frogs depends on the degree of evolutionary divergence between the source and target species, with the success rate and polymorphism declining rapidly with increasing divergence between the taxa. However, given the relatively high levels of microsatellite polymorphism in some of the target species, these markers may provide useful tools for future conservation genetic studies aiming to address taxonomic uncertainties, or to study genetic variability and differentiation of *Indirana* populations.

## Competing interests

Authors declare that they have no competing interests.

## Author contributions

AN, SG and JM conceived the study. SVG, SG and KSK collected all the samples. AN performed all the experiments, analyses and had a major role in writing the manuscript together with JM. All authors contributed to and approved the final manuscript.

## Supplementary Material

Additional file 1 **Cross species amplification of microsatellite loci in eight species from the*Indirana*genus.** A = number of alleles observed, n = number of individuals tested. A dash indicates no amplification success. (XLSX 15 kb) Click here for file
